# The Role of the Heterogeneous Catalyst to Produce Solketal from Biodiesel Waste: The Key to Achieve Efficiency

**DOI:** 10.3390/nano14100828

**Published:** 2024-05-09

**Authors:** Catarina N. Dias, Alexandre M. Viana, Luís Cunha-Silva, Salete S. Balula

**Affiliations:** LAQV/REQUIMTE & Department of Chemistry and Biochemistry, Faculty of Sciences, University of Porto, 4169-007 Porto, Portugal; up201804900@edu.fc.up.pt (C.N.D.); up201405091@edu.fc.up.pt (A.M.V.); l.cunha.silva@fc.up.pt (L.C.-S.)

**Keywords:** glycerol, acetalization, solketal, heterogeneous catalysts

## Abstract

The valorization of the large amount of crude glycerol formed from the biodiesel industry is of primordial necessity. One possible direction with high interest to the biorefinery sector is the production of fuel additives such as solketal, through the acetalization of glycerol with acetone. This is a chemical process that conciliates high sustainability and economic interest, since solketal contributes to the fulfillment of a Circular Economy Model through its use in biodiesel blends. The key to guarantee high efficiency and high sustainability for solketal production is the use of recovery and recyclable heterogeneous catalysts. Reported works indicate that high yields are attributed to catalyst acidity, mainly the ones containing Brönsted acidic sites. On the other hand, the catalyst stability and its recycling capacity are completely dependent of the support material and the acidic sites incorporation methodology. This review intends to conciliate the information spread on this topic and indicate the most assertive strategies to achieve high solketal production in short reaction time during various reaction cycles.

## 1. Introduction

**Figure 1:**
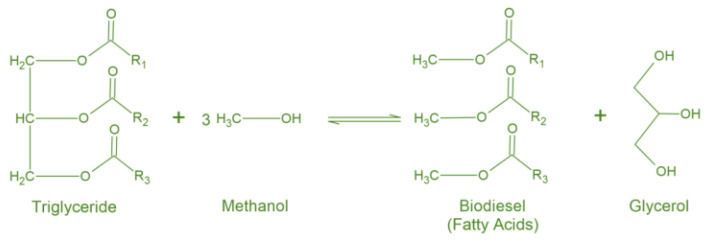
Transesterification reaction of triglycerides in the presence of methanol, originating biodiesel and glycerol as a by-product [10].

The development of global civilization has progressively increased energetic demands through history. In order to answer these needs, fossil fuel production and consumption soared throughout the last century, with consistently rising demand for coal, oil, and natural gas as the most predominant energy sources. However, a shift towards production and consumption of other energy sources is a major societal challenge due to the negative environmental impact of fossil fuels and their unrenewable supply [1,2]. Fossil fuels are responsible for most of the carbon dioxide emissions, but are also composed of various sulfur- and nitrogen-containing compounds which directly contribute to greenhouse gas (GHG) emissions [3,4]. The growing understanding and awareness of climate change has led to the implementation of legislation to regulate fuel production and environmental policies to drive the development and adoption of new energy alternatives for a more sustainable development [5]. However, nowadays fossil fuel sources relate to approximately 81% of today’s global energy consumption and global demand is projected to peak in 2030. Even though oil demand in advanced economies peaked back in 2005 and its expected to decline further in the coming decade, it is projected to continually grow in other regions until 2050 [6,7]. Fossil fuels are constituted by various sulfur and nitrogen constituents that when burned originate oxides (SO_x_ and NO_x_), releasing harmful emissions (Figure 1). Furthermore, fossil fuels are also one of the main direct contributors of greenhouse gases (GHGs), responsible for most carbon dioxide emissions [3,8]. Consequently, the European Union has planned to reduce 55% of GHG emissions by 2030, and completely eliminate them by 2050 [9].

Biodiesel is considered a non-toxic, biodegradable, and renewable fuel, allowing for safer handling and reduced carbon, sulfur, and particulate matter emissions. Further, when compared to normal fuels, it exhibits a higher cetane number and better lubrication, contributing to a better engine performance and fuel consumption efficiency [3,5]. However, pure biodiesel also demonstrates some drawbacks, related to its higher viscosity and less satisfying cold properties, which could lead to serious problems in engine longevity. Therefore, biodiesel is normally blended with fuel additives, whose application helps solve these problems and, therefore, benefit biodiesel commercialization [2,10]. Biodiesel is obtained through the transesterification reaction of triglycerides (a type of fat found in vegetable oils) with a short-chain alcohol such as methanol (Figure 1), in the presence of an appropriate catalyst and under acidic or basic conditions [1,2,10]. It is also known as fatty acid alkyl esters (FAAEs) or fatty acid methyl esters (FAMEs), depending on the alcohol used in the reaction (ethanol or methanol, respectively) [1,11].

**Figure 2:**
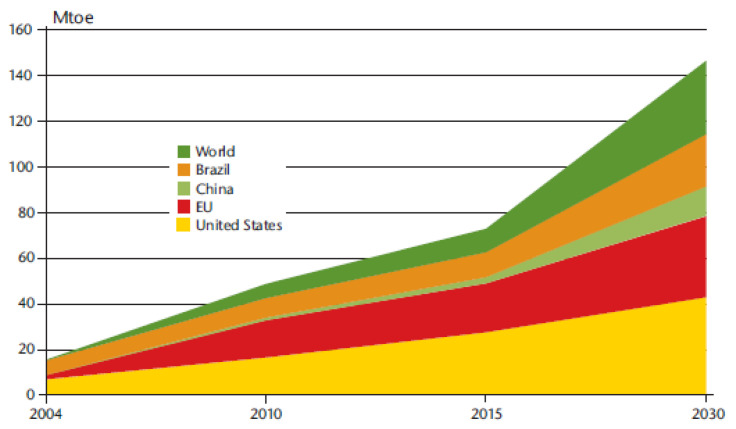
Development of world biodiesel consumption [17].

Propane-1,2,3-triol (C_3_H_8_O_3_), also known as glycerol or glycerin, is the by-product of this reaction, originated as 10% in weight in relation to biodiesel. As such, there is an excess in crude glycerol in the fuel industry, owing to the increased interest in biodiesel production [12,13,14,15]. As of 2022, global biodiesel production amounted to approximately 53,000 million liters and the latest projection of the Organisation for Economic Co-operation and Development gives an increase of 25% in global production until 2032 (Figure 2) [16,17]. In order to increase biodiesel’s own sustainability, it is extremely important to develop strategies capable of reutilizing crude glycerol obtained as waste.

**Figure 3:**
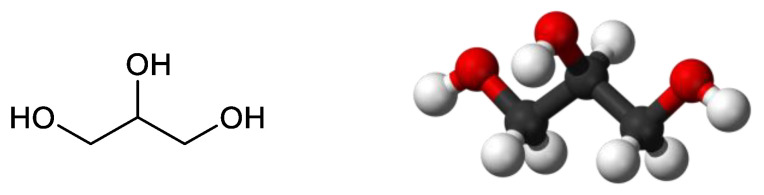
Molecular structure of glycerol.

Glycerol is a compound with a high boiling point, low volatility, and low toxicity, constituted by three hydroxyl substituents which allow for the formation of hydrogen bonds. These bonds are responsible for its solubility in water and its high viscosity (Figure 3) [11,12]. Glycerol finds application in various industries, from cosmetics and pharmaceuticals to the food industry, but is also a compound of interest as a chemical platform for conversion into value-added products [11,13].

After transesterification, it is necessary to follow several steps in order to efficiently obtain the produced biodiesel: (i) neutralization of the reaction mixture, owing to the acidic or basic nature of the catalyst, (ii) removal of the unreacted methanol, that was used in excess, through distillation, and (iii) separation of biodiesel from glycerol and other substances [2,5,18]. As such, the final glycerol, i.e., crude glycerol, possesses impurities related to these stages, which can vary according to the raw source used, the efficiency of the washing and biodiesel separation procedures, among other factors. The most common impurities found in crude glycerol are water, salts, ash, and methanol [18,19].

**Figure 4:**
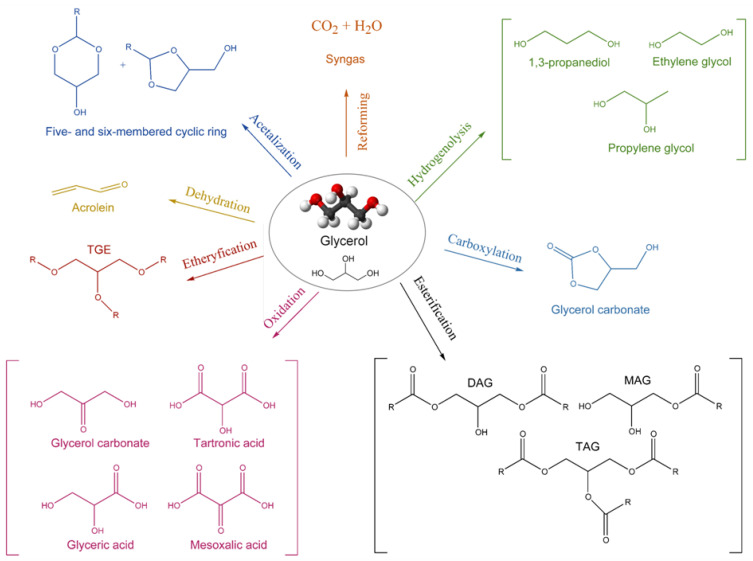
Glycerol valorization reactions and the respective products obtained. Adapted from the reference [21].

As mentioned previously, glycerol has many different uses, but it is important to take into consideration that most are only effective using pure glycerol. The conversion of glycerol into added-valuable products can only be achieved by performing a previous purification of crude glycerol; otherwise, the efficiency of the process can be compromised [3,14,20]. On the other hand, the isolate process of glycerol purification is not an attractive economic method, since this is extremely costly, and therefore, not economically viable [3,19]. As such, the most commonly adopted strategy is glycerol valorization, where a variety of catalytic reactions were found to be able to transform glycerol. Figure 4 illustrates the most utilized pathways.

Hydrogenolysis of glycerol occurs in a catalytic system combining dehydration and hydrogenation processes [22,23,24,25]. In general, glycerol undergoes dehydration in the presence of an acidic catalyst, followed by the addition of a hydrogen source, commonly using transition metals. The most commercially interesting products originated by this reaction are 1,2-propanediol and 1,3-propanediol, whose applications range from pharmaceuticals to cosmetics and, most commonly, polymer formulation [13,22]. Oxidation of glycerol can originate a wide variety of products, depending on the nature of the catalyst used and the reaction environment [26,27,28,29]. The most well-known products are glyceric acid and dihydroxyacetone, obtained when the oxidation occurs in a primary or secondary hydroxyl group, respectively. Applications range from pharmaceuticals and cosmetics to use as protective agents in coatings [23,26]. Dehydration of glycerol in the presence of catalysts with an acidic nature, such as Brönsted or Lewis acids, originates acrolein. This compound is used as an intermediate for many other products, such as acrylic acid, mostly for polymer formulation [30,31]. The etherification reaction of glycerol originates fuel additives, such as di-ethers and tri-ethers, in the presence of acid or basic catalysts [32,33,34]. Other pathways can be reduction, carboxylation, oligomerization, and pyrolysis [35].

Acetalization is one of the most promising glycerol valorization methodologies producing valuable products by clean and moderate procedures. The acetalization/ketalization of glycerol has been studied under different conditions and catalysts. The cyclic acetals and ketal products obtained with aldehydes and ketones, respectively, present a large variety of applications [36]. One of the most desired is solketal, being a renewable raw material, obtained from glycerol by an acid-catalyzed reaction with acetone [12]. Solketal presents a high variety of applications. As a fuel additive, it reduces fuel gelling, particles emission, and fuel consumption. The use of solketal with gasoline enhances the octane number [37]. Furthermore, solketal presents other useful applications such as a solvent in the paint and ink industries, and as a component in pharmaceuticals, cosmetics, and polymer chemistry, including the development of drug delivery materials [38]. However, studies demonstrating the viability of solketal’s industrial applications are scarce [39].

This review intends to conciliate the information presented in the literature about the production of solketal from glycerol with acetone. The most important parameters that can influence its fast and selective preparation will be identified and discussed, giving a stronger emphasis to the catalyst nature and structure, since this is the main key to obtain solketal from crude glycerol. The use of a most suitable catalyst promotes a green route to prepare solketal at low temperature, even at room temperature, under a solvent-free system [10,40].

## 2. Acetalization Reaction: Parameters That Can Influence Efficiency

**Figure 5:**
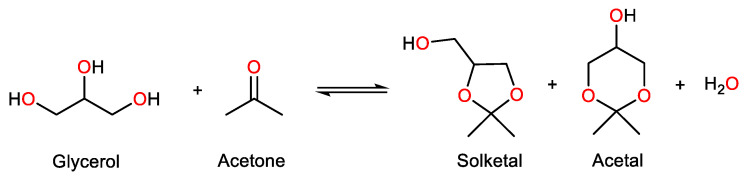
Acetalization reaction of glycerol, in the presence of acetone, originating solketal, acetal, and water [10].

The glycerol acetalization reaction takes place in the presence of aldehydes or ketones, originating a five-membered cyclic compound and a six-membered cyclic compound [11,19]. Finally, water is obtained as a by-product of the acetalization reaction. When in the presence of acetone (Figure 5), the reaction product obtained with highest selectivity is 2,2-dimethyl-1,3-dioxolane-4-methanol (C_6_H_12_O_3_), commonly known as solketal. This compound is considered environmentally friendly, combining low toxicity with high miscibility in most solvents, which favors its application in various industries [3,10]. However, the use of solketal as an oxygenated fuel additive is extremely interesting, especially when applied to biodiesel blends. As mentioned previously, biodiesel cannot be used in its pure form, since its high viscosity and under-performing cold flow properties can become a very serious problem for engine functioning, and the high NO_x_ emissions raise an environmental concern. However, when biodiesel is blended, i.e., combined with fuel additives, these problems are eliminated, since these substances have the ability to improve fuel characteristics [13,20]. Amongst many other things, additives can decrease the viscosity of the fuel, act as cleanliness agents, and provide a shorter ignition delay, which prevents unnecessary particulate matter and NO_x_ emissions [10,12,19]. Further, the use of solketal as a fuel additive for biodiesel is economically advantageous, allowing the application of a Circular Economy perspective [11], since (i) the biodiesel formation reaction originates glycerol as a by-product, creating an overplus, (ii) through acetalization, glycerol can be repurposed as solketal, whose interest as a fuel additive has been suggested, and (iii) biodiesel requires the use of fuel additives to be commercialized.

The acetalization of glycerol is a reversible reaction, hindered by the existence of a large thermodynamic setback owing to its low equilibrium constant [10,20]. Further, this reaction originates water as a by-product, whose presence has been proven to greatly decrease the solketal yield obtained [3,11,19]. As such, it is essential to adopt strategies that guarantee that the reaction is shifted in favour of the products, while assuring optimal conditions for solketal formation. This reaction efficiency is linked to the correct choice of the substrate, solvent, and catalyst.

### 2.1. Substrate

**Figure 6:**
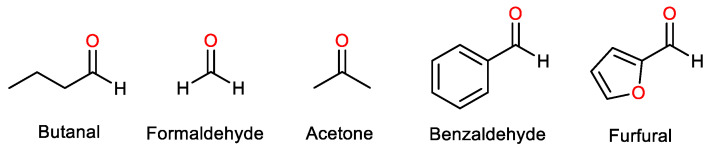
Examples of substrates used in the acetalization reaction of glycerol, mainly aldehydes (formaldehyde, benzaldehyde, butanal, furfural) and ketones (acetone).

One of the most adopted strategies to increase glycerol conversion is to use a substrate in excess, increasing the glycerol/substrate ratio. In acetalization, substrates are oxygen-containing compounds, such as aldehydes and ketones (Figure 6). Many different substrates have been used in acetalization reactions before, with the most reported ones being butanal [41], furfural [42], citral [43], benzaldehyde [44], formaldehyde [45], and acetone. This review will be focused on acetone, as it is by far the most studied substrate, and its application in glycerol conversion has proved incredibly effective [46,47,48]. Further, excess acetone has been reported to increase glycerol conversion to solketal, while also acting as an entrainer, helping the removal of water from the reactional system, and increasing its miscibility with the viscous glycerol [10,12]. When the reaction is finalized, the unreacted acetone can be recuperated through distillation and be continuously reutilized [12], which helps ensure the sustainability of the acetalization reaction.

### 2.2. Solvent

As seen previously, when glycerol undergoes, acetalization, it originates two other products besides solketal: acetal and water. Removal of water can be ensured by the use of entrainers, desiccants and membranes, amongst other methods [10,49]. The removal of acetal helps shift the reaction in order to obtain higher glycerol conversions, while simultaneously allowing its recuperation. Further, while solketal is a product of higher commercial interest, acetal also demonstrates fuel additive qualities, and therefore should not be wasted. Traditionally, the removal of acetal from the reactional system was possible through the use of solvents. Some examples that have been previously reported in the literature include toluene [50], ethanol [51], and acetonitrile [52]. The evolution of research in the last years allowed the development of highly efficient catalysts that can assure a favourable reaction equilibrium by themselves. As such, acetalization reactions have evolved into solvent-free environments [12,46,53,54,55].

### 2.3. Catalyst

**Figure 7:**
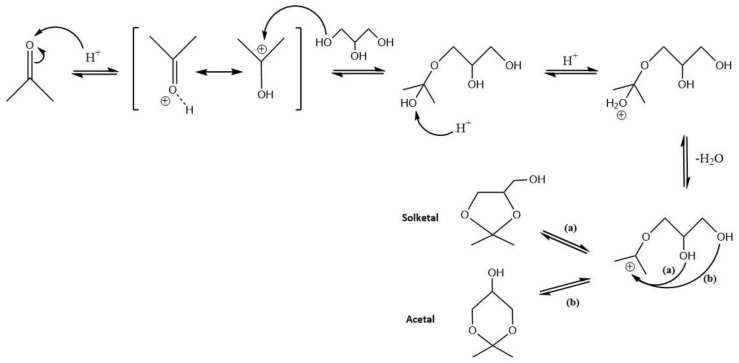
Mechanism of the acetalization reaction of glycerol, with acetone as the substrate and in the presence of a Brönsted acid catalyst, illustrating the formation of the five- and six-membered cyclic products, solketal and acetal, respectively.

In glycerol acetalization, the correct choice of the catalyst is one of the most important reaction parameters since, without the presence of a catalyst, the reaction practically does not occur and no glycerol conversion can be observed [56,57,58,59]. The importance and the role of catalysts in this reaction becomes clear when observing the reaction mechanism behind glycerol acetalization. According to previous speculation, a proposed mechanism can be seen in Figure 7, in this case specifically for a Brönsted acid catalyst [19]. In general, the reaction is kickstarted when the catalyst interacts with the carbonyl of the substrate, either by protonation or coordination with a metal site (for Brönsted and Lewis acids, respectively) [40,46,47,60]. This interaction forms a protonated intermediate structure that, when interacting with the hydroxyl groups in glycerol, originates a hemiketal/hemiacetal. Once the water molecules are removed from the reaction, the formation of a tertiary carbenium ion occurs [12,19]. Finally, this structure suffers an attack from the hydroxyl groups from glycerol and solketal is originated from the interaction of the ion with a secondary −OH, and acetal occurs from the interaction with a primary −OH [56,57,61]. As such, product selectivity for solketal is much higher, and as a consequence the attack of the secondary hydroxyl is more facilitated, since the primary −OH suffers steric hindrance [3,10,55].

Essentially, the role of the catalyst is to assure the activation of the substrate and initiate the acetalization of glycerol. Further, it has been extensively reported that the efficiency of this initial activation, and thus the efficiency of glycerol conversion, is highly dependent on catalyst acidity [40,57,61,62]. In Section 3 the various catalysts that have been used since 2012 for the acetalization of glycerol with acetone to form solketal without using auxiliary solvents will be presented. A careful discussion is here presented correlating the nature and structure of catalysts and their efficiency and stability.

## 3. Heterogeneous Catalysts for Glycerol Acetalization

Conventionally, acetalization reactions required the use of homogeneous catalysts, such as sulfuric acid, hydrochloric acid, and p-toluenesulfonic acid [10,12]. However, the use of these catalysts lead to various reaction drawbacks requiring long reaction times and exhibiting difficult recuperation from the reaction medium, which increased the cost [12]. Further, and most importantly, these catalysts are known for their environmental problems, raising attention for their toxicity [3,10]. The awareness for reaction sustainability and its alignment with the Principles of Green Chemistry raised interest in the search for alternative catalysts that allowed high catalytic efficiency and recyclability, while facilitating handling/recovery and being environmentally friendly [3,20,63]. As such, heterogeneous catalysts appeared as potential candidates for the acetalization reaction of glycerol [10,12]. In the last years, many different catalysts and their application in glycerol conversion have been reported, with some examples being heteropolyacids, mesoporous silicas, metal–organic frameworks (MOFs), resins, carbon-based materials, and polymers. Table 1, Table 2, Table 3 and Table 4 present various reported glycerol conversion and solketal selectivity results, using different types of heterogeneous catalysts, in the acetalization of glycerol using acetone under solvent-free systems.

**Figure 8:**
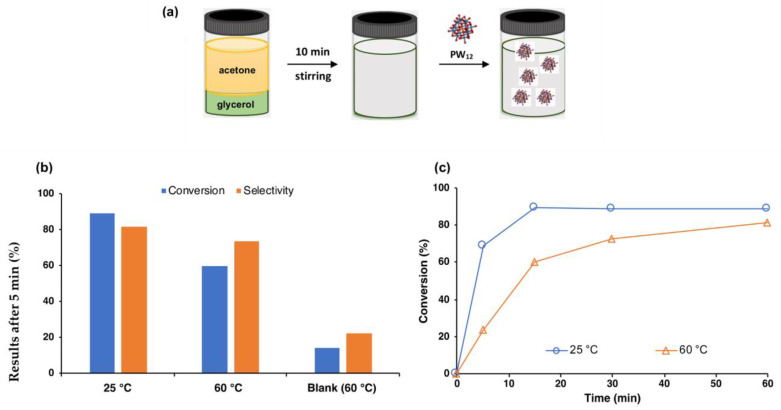
(**a**) Schematic representation of the catalytic reaction scheme; (**b**) conversion and solketal selectivity obtained at different temperatures (25 °C and 60 °C) and blank reaction at 60 °C, obtained after 5 min of reaction; (**c**) catalytic profile for glycerol acetalization at 25 °C and 60 °C. All results were obtained using a glycerol/acetone ratio of 1:6 and PW_12_ (3% referred to glycerol weight) as catalyst. Adapted from reference [46].

Balula et al. studied the influence of Keggin-type heteropolyacids, with the use of phosphotungstic acid (PW_12_), phosphomolybdic acid (PMo_12_), and silicotungstic acid (SiW_12_), in the acetalization reaction of glycerol at room temperature (Figure 8) [46]. The results reported a catalytic efficiency trend of PW_12_ (99.2%) > PMo_12_ (91.4%) > SiW_12_ (90.7%) after only 10 min, where PW_12_ is widely reported to be the most acidic out of the three heteropolyacids [46,64,65]. Da Silva et al. developed a cation-exchanged heteropolyacid, where the protons of silicotungstic acid were substituted by tin(II) cations [56]. Such a change assured heteropolyacid salt insolubility, in an effort to solve the recuperation problems associated with this type of catalyst [64,66]. Glycerol conversion reached 99% after 1 h, with high selectivity at room temperature, owing to the characteristic acidic behaviour of Sn_2_SiW_12_O_40_, with the catalyst possessing both Brönsted and Lewis acid sites [56]. The catalyst was reused for four consecutive cycles, demonstrating catalytic stability; however, catalyst recuperation was very burdensome [56]. Also, cationic exchange was performed by Ali et al. using imidazolium cations; however, the conversion and selectivity of the glycerol acetalization was not increased when compared with the commercial acids of polyoxometalates [67]. Chen et al. investigated another possibility of facilitating heteropolyacids as catalysts in acetalization, through the preparation of a cesium phosphotungstic salt, and its consequent immobilization in KIT-6 silica [58]. The conversion results obtained for the catalyst in its bulk and incorporated form were very similar (94 and 95%, respectively), with Cs_2.5_H_0.5_PW_12_O_40_@KIT-6 reaching higher conversions after only 15 min. Stability tests showed no loss of activity after three consecutive cycles, demonstrating its effectiveness [58].

**Table 1:** Metallic oxide-based catalysts used for glycerol acetalization reactions, with acetone as a substrate and in the absence of an auxiliary solvent.

Catalyst	Ratio of Glycerol/Acetone	Temperature (°C)	Time (h)	Conversion (%)	Selectivity to Solketal (%)	Ref.
H_3_PW_12_0_40_	1:15	RT	0.08	99.2	97	[46]
H_3_PMo_12_0_40_	1:15	RT	0.08	91.4	94	[46]
H_4_SiW_12_O_40_	1:15	RT	0.08	90.7	85.7	[46]
Sn_2_SiW_12_O_40_	1:16	RT	1	99	97	[56]
Cs_2.5_H_0.5_PW_12_O_40_	1:6	RT	1	94	98	[58]
Cs_2.5_H_0.5_PW_12_O_40_@KIT-6	1:6	RT	0.25	95	98	[58]
meso-MoO_2_	1:10	RT	1	95.8	97.8	[68]
meso-WO_3_	1:10	RT	1	34.7	71.2	[68]
meso-SnO_2_	1:10	RT	1	28.9	68.9	[68]
SnO_2_	1:1	RT	1.5	15	96	[47]
WO_3_/SnO_2_	1:1	RT	1.5	55	90	[47]
MoO_3_/SnO_2_	1:1	RT	1.5	61	96	[47]
SO_4_^2−^/SnO_2_	1:1.5	RT	4	98	96	[69]
MoO_3_-ZrO_2_	1:8	50	0.2	89	97	[70]
[HMIm]_3_[PW_12_O_40_]	1:2	RT	1	85	87.06	[67]
[HMIm]_3_[PMo_12_O_40_]	1:2	RT	1	80	82.5	[67]
[HMIm]_4_[SiW_12_O_40_]	1:2	RT	1	76	78.94	[67]

Mallesham et al. prepared modified SnO_2_ catalysts, whose catalytic performance was studied in the acetalization reaction at room temperature [47,69]. After 1 h, the following results were obtained: SO_4_^2−^/SnO_2_ (98%) > MoO_3_/SnO_2_ (61%) > WO_3_/SnO_2_ (55%). All three catalysts exhibited higher conversion results than the non-modified SnO_2_ solid acid (15% after 1.5 h), with SO_4_^2−^/SnO_2_ demonstrating superior conversion owing to the presence of super acidic sites in its structure, further confirming the influence of catalyst acidity [69]. In summary, of the metallic oxide basic catalysts, the polyoxometalates showed a higher conversion rate and higher selectivity for solketal production.

Among the various heterogeneous catalysts based on silica (Table 2) used for the acetalization of glycerol with acetone, the work from Gadamsetti et al. [55] presented one of the best catalytic results. In this case, the development of a silica-incorporated molybdenum phosphate catalyst is reported, and its consequent study in acetalization of glycerol, at room temperature. The prepared catalyst demonstrated perfect glycerol conversion, combined with high solketal selectivity (98%), after only 1 h. Through material characterization, it was shown that MoPo@SBA-15 possessed Brönsted acidic sites, responsible for the high glycerol conversion. Catalyst stability was evaluated for four consecutive recycling cycles, demonstrating the existence of acidic sites leaching, corresponding to a decrease in catalytic efficiency [55]. Another important catalytic achievement was achieved by Ammaji et al. by incorporating transition metals in the SBA-15 structure, further studying the application of SBA-15-based catalysts in acetalization reactions [57]. At room temperature, the follow order of conversion capacity was obtained: Nb-SBA-15 (95%) > Zr-SBA-15 (92%) > Ti-SBA-15 (65%) > Al-SBA-15 (60%), with the Nb-SBA-15 catalyst demonstrating the best catalytic results, along with complete solketal selectivity [57]. Similarly to previous reports, the best-performing catalysts (Nb-SBA-15 and Zr-SBA-15) were those that exhibited the highest amount of Brönsted acidic sites, highlighting its importance for this particular reaction. The Nb-SBA-15 catalyst was continuously applied in acetalization reactions for four cycles, showing a decrease in glycerol conversion which was related to leaching of acidic sites [57]. Comparing in general the catalytic results obtained with the functional silica catalysts (Table 2) with the previous heterogeneous polyoxometalates (Table 1), it is possible to observe that identical results were obtained for solketal conversion and selectivity, with shorter reaction times (0.08 or 0.25 h) when polyoxotungstates were used and in the presence of a lower ratio of glycerol/acetone (1:3) when Nb-SBA-15 or Zr-SBA-15 were used. 

**Table 2:** Silica-based catalysts used for glycerol acetalization reactions, with acetone as a substrate, under a solvent-free system.

Catalyst	Ratio of Glycerol/Acetone	Temperature (°C)	Time (h)	Conversion (%)	Selectivity to Solketal (%)	Ref.
MoPo@SBA-15	1:3	RT	1	100	98	[55]
Nb-SBA-15	1:3	RT	1	95	100	[57]
Zr-SBA-15	1:3	RT	1	92	98	[57]
Ti-SBA-15	1:3	RT	1	65	98	[57]
Al-SBA-15	1:3	RT	1	60	98	[57]
Ar-SBA-15	1:6	70	0.5	82.5	wi	[71]
Pr-SBA-15	1:6	70	0.5	79.0	wi	[71]
PSF	1:10	RT	1.5	75	98	[72]
PSF/SiO_2_	1:10	RT	1.5	86.6	98	[72]
PSF/K-SiO_2_	1:10	RT	1.5	86.3	98	[72]
MoO_3_/SiO_2_	1:2	RT	1	46.8	90	[59]
SO_4_-Al-MCM-41	1:10	RT	2	94.8	99	[73]

wi: without information.

**Figure 9:**
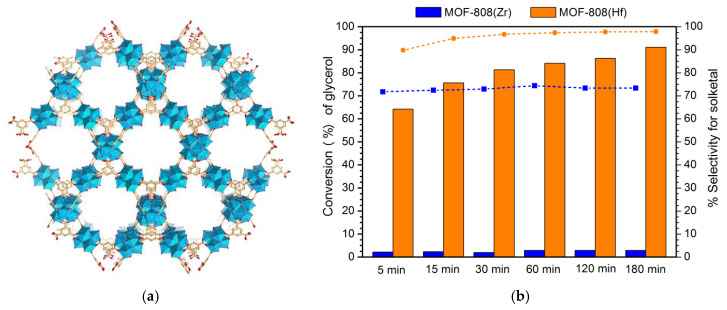
(**a**) Structure of MOF-808 (Zr); (**b**) Conversion of glycerol by acetalization reaction catalyzed by MOF-808 (Zr) and MOF-808 (Hf) materials (15 mg) using a ratio of 1:6 glycerol/acetone and a temperature of 60 °C.

Carbon-based materials, such as metal–organic frameworks (MOFs), have also been used as heterogeneous catalysts for the acetalization of glycerol with acetone (Table 3). Among these works, Bakuru et al. [61] presented one of the most active and sustainable catalytic systems based on MOFs. In this case, the effect of acidity in the structure of UiO-66 was studied, and its influence in the acetalization of glycerol, at room temperature. This MOF structure is very interesting for acetalization, since the combination of the oxophilicity behavior and the existence of defects causes the appearance of more acidic sites in its structure [61]. From the three MOFs studied, it was seen that UiO-66 (Hf) (94.5%) > UiO-66 (Ce) (70.9) > UiO-66 (Zr) (1.5%), confirming that UiO-66 (Hf) is the best-performing catalyst since it has the highest amount of µ_3_-OH groups, acting as Brönsted acidic sites [74]. The higher the oxophilicity of the MOF structure, the higher the acidity, which originates a higher glycerol conversion [61]. Mirante et al. compared the catalytic efficiency of another family of MOFs, based on MOF-808 [40]. Similarly, MOF-808 (Hf) exhibited the best catalytic behaviour, reaching 91% after 3 h at 60 °C, which was expected due to the superior acidity obtained when compared to the MOF-808 (Zr) catalyst (Figure 9). Catalyst recycling was evaluated for ten consecutive cycles, with MOF-808 (Hf) demonstrating high stability [40]. Santos-Vieira et al. reported the preparation of a coordination polymer (UAV-59), constituted by Gd^3+^ cations and nitrile (trimethylphosphonic acid) [60]. This catalyst was applied to acetalization reactions, at 55 °C, obtaining a glycerol conversion of 94%, with simultaneous high solketal selectivity (97%). The efficiency of this polymer can be explained by the high concentration of acidic protons in its structure. Catalyst stability studies demonstrated only a minor decrease in activity, after four consecutive recycling cycles [60]. The best catalytic performance between the MOF-based materials presented in Table 3 is shown by a composite formed by the incorporation of a polyoxotungstate into the MOF–Fe framework [HMIm]_3_[PW_12_O_40_]@MOF-Fe (Table 3) [67]. This catalyst obtained complete glycerol conversion and complete solketal selectivity, after only one hour, using the lowest ratio of glycerol/acetone reported in the literature. Using [HMIm]_3_[PW_12_O_40_]@MOF-Fe catalyst during seven recycling cycles, the glycerol conversion and solketal selectivity were maintained, demonstrating the superior acetalization behaviour of this catalyst compared to the isolated polyoxotungstate [67].

**Table 3:** MOF-based catalysts used for glycerol acetalization reactions, with acetone as a substrate and in the absence of an auxiliary solvent.

Catalyst	Ratio of Glycerol/Acetone	Temperature (°C)	Time (h)	Conversion (%)	Selectivity to Solketal (%)	Ref.
UiO-66 (Hf)	1:4	RT	1	94.5	97.2	[61]
UiO-66 (Ce)	1:4	RT	1	70.9	90.1	[61]
UiO-66 (Zr)	1:4	RT	1	1.5	73.2	[61]
UiO-SO_3_H-0.5	1:10	60	1	60.2	99.7	[48]
MOF-808 (Hf)	1:6	60	3	91	98	[40]
MOF-808 (Zr)	1:6	60	3	6	100	[40]
MOF-Fe	1:2	RT	1	72	72.22	[67]
MIL-118 (Al)	1:10	wi	4	43	58	[75]
MIL-118-SnO_2_	1:10	wi	4	76	97	[75]
UAV-59	1:10	55	2	94	97	[60]
UAV-63	1:10	55	6	84	96	[76]
UAV-20	1:10	55	6	56	90	[76]
[HMIm]_3_[PW_12_O_40_]@MOF-Fe	1:2	RT	1	100	100	[67]
[HMIm]_3_[PMo_12_O_40_]@MOF-Fe	1:2	RT	1	95	96.84	[67]
[HMIm]_4_[SiW_12_O_40_]@MOF-Fe	1:2	RT	1	90	93.33	[67]
PVA40	1:6	70	3	94	wi	[77]
SCS1/2	1:6	70	0.5	75	90	[78]
HSCS1/2	1:6	70	0.5	82	99	[78]
SO_3_H-C	1:8	57	1	80	wi	[79]

wi: without information.

Few reported works can also be found in the literature using zeolite-based heterogeneous catalysts for acetalization of glycerol with acetone (Table 4). Using this type of catalyst, a higher ratio of glycerol/acetone needed to be used to achieve similar results to those obtained with MOFs and polyoxotungstate (Table 3). One of the most interesting examples is reported by Saini et al., who developed a metal-free mordenite zeolite catalyst which was applied in acetalization reactions at 60 °C [80]. After 4 h of reaction, the catalyst obtained 99% of glycerol conversion, while demonstrating high solketal selectivity (99%). Mordenite was recycled for three cycles, showing no loss of activity [80]. Other interesting kinetic studies using Amberlyst-35 catalyst are also presented in the literature [81]. However, in this study an auxiliary solvent was used.

**Table 4:** Zeolite-based catalysts and other composites used for glycerol acetalization reactions, with acetone as a substrate and in the absence of an auxiliary solvent.

Catalyst	Ratio of Glycerol/Acetone	Temperature (°C)	Time (h)	Conversion (%)	Selectivity to Solketal (%)	Ref.
ZrMo-KIT-6	1:8	50	4	85.8	97.8	[82]
Zeolite HY	1:2	RT	1	74.2	98.2	[59]
Zeolite OTS-HY	1:12	30	1	89	95	[83]
Zeolite H-Beta-1	1:2	RT	1	86	98.5	[59]
Zeolite HBEA	1:10	RT	1.5	70.9	97.5	[72]
Zeolite Mordenite	1:10	60	4	99	99	[80]
Amberlyst-15	1:2	RT	1	73.1	91	[59]
Amberlyst-45	1:10	RT	1.5	80.6	97.4	[72]

## 4. Conclusions

The implementation of legislation designed to reduce and eventually eliminate completely the use of fossil fuels derivatives has demonstrated the increasingly urgent search for new sustainable energy sources. Biodiesel has been explored as a non-toxic and environmentally friendly alternative, whose formation reaction still needs to be optimized in order to increase its sustainability and economic interest. An obstacle associated with this industry is the large amount of glycerol produced as a by-product, raising importance for the discovery and investigation of glycerol valorization strategies, such as acetalization. The acetalization reaction of glycerol, in the presence of acetone, originates solketal, a very interesting fuel additive that contributes to the fulfilment of a Circular Economy Model through its use in biodiesel blends. In the last years, heterogeneous catalysts have distinguished themselves in acetalization, allowing high conversion and selectivity, while simultaneously facilitating recuperation and increasing the sustainability of this valorized process. High glycerol conversion results are linked to the catalyst acidity, where the preference of Brönsted acidic sites over Lewis sites has been extensively reported, owing to their efficient activation of the substrate, and thus increasing its interaction with glycerol and producing higher amounts of solketal. The acetalization of glycerol has been confirmed to be a fast-acting reaction, using mild experimental conditions, since the majority of works published report better conversion results at room temperature. Higher temperatures, such as 55 °C or 60 °C, are also verified, mainly in order to increase acetone/glycerol miscibility and increase diffusion. Several excellent results have been obtained thus far; however, there is still much room for improvement, for example: (i) mesoporous silicas demonstrate high conversion results at fast rates, but many report stability issues linked with leaching of the acidic sites; (ii) the application of MOFs has raised interest, owing to the combination of their satisfactory glycerol conversion and recyclability behaviour. Another advantage of using silica- and MOF-based materials is the lower ratio of glycerol/acetone needed to achieve near complete conversion and 100% of selectivity for thee solketal after 1 h of reaction. Keggin-type polyoxotungstates have been shown to achieve the same catalytic results but with shorter reaction times, such as after 10 and 20 min. However, the combination of the most promising catalysts, i.e., the acid polyoxometalates and MOFs or silicas, for the production of solketal is practically unexplored, and the only example reported is considered the most sustainable and productive for this acetalization reaction. Advanced catalysts for solketal production will be needed in the future and these should be designed using acid polyoxometalates incorporated into silicas and/or MOFs.

## Data Availability

Not applicable.
